# Giving the prostate the boost it needs: Spiral diffusion MRI using a high‐performance whole‐body gradient system for high *b*‐values at short echo times

**DOI:** 10.1002/mrm.30351

**Published:** 2024-11-04

**Authors:** Malwina Molendowska, Lars Mueller, Fabrizio Fasano, Derek K. Jones, Chantal M. W. Tax, Maria Engel

**Affiliations:** ^1^ Cardiff University Brain Research Imaging Centre (CUBRIC) Cardiff University Cardiff UK; ^2^ Medical Radiation Physics, Clinical Sciences Lund Lund University Lund Sweden; ^3^ Leeds Institute of Cardiovascular and Metabolic Medicine University of Leeds Leeds UK; ^4^ Siemens Healthcare Ltd Camberly UK; ^5^ Siemens Healthcare GmbH Erlangen Germany; ^6^ Image Sciences Institute, University Medical Center Utrecht Utrecht The Netherlands

**Keywords:** data acquisition, diffusion, field monitoring, image reconstruction, microstructure, prostate cancer

## Abstract

**Purpose:**

To address key issues of low SNR and image distortions in prostate diffusion MRI (dMRI) by means of using strong gradients, single‐shot spiral readouts and an expanded encoding model for image reconstruction.

**Methods:**

Diffusion‐weighted spin echo imaging with EPI and spiral readouts is performed on a whole‐body system equipped with strong gradients (up to 250 mT/m). An expanded encoding model including static off‐resonance, coil sensitivities, and magnetic field dynamics is employed for image reconstruction. The acquisitions are performed on a phantom and in vivo (one healthy volunteer and one patient with prostate cancer). The resulting images are compared to conventional dMRI EPI with navigator‐based image reconstruction and assessed in terms of their congruence, SNR, tissue contrast, and quantitative parameters.

**Results:**

Using the expanded encoding model, high‐quality images of the prostate gland are obtained across all *b*‐values (up to 3 ms/μm^2^), clearly outperforming the results obtained with conventional image reconstruction. Compared to EPI, spiral imaging provides an SNR gain up to 45% within the gland and even higher in the lesion. In addition, prostate dMRI with single‐shot spirals at submillimeter in‐plane resolution (0.85 mm) is accomplished.

**Conclusion:**

The combination of strong gradients and an expanded encoding model enables imaging of the prostate with unprecedented image quality. Replacing the commonly used EPI with spirals provides the inherent benefit of shorter echo times and superior readout efficiency and results in higher SNR, which is in particular relevant for considered applications.

## 1 INTRODUCTION

Diffusion MRI (dMRI) signals carry information about the motion of water molecules, which is modulated by the microstructure of the tissue.^1^, ^2^, ^3^ By manipulating the contrast‐generating magnetic‐field gradients, descriptors of tissue properties at macroscopic and microscopic scales can be inferred. Yet, the inherently low SNR of dMRI data and presence of image artifacts diminish the accuracy and discriminative power of associated quantitative parameter maps.

Several developments on the hardware, acquisition, and reconstruction side have been proposed to improve the reliability of dMRI. Recent hardware developments include novel whole‐body and head‐only MR systems with ultra‐strong gradients,^4^, ^5^, ^6^, ^7^, ^8^ which enable a shorter TE for a given *b*‐value, and hence a higher SNR per unit time. These MR machines have greatly advanced the characterization of the microstructure of human tissue in vivo, albeit to date almost exclusively limited to the brain.^9^, ^10^, ^11^, ^12^, ^13^, ^14^, ^15^ Yet, these machines also hold great promise for the advancement of microstructural MRI in parts of the body other than the brain,^16^ such as prostate^17^, ^18^ and heart.^19^ However, strong gradients come with a set of caveats such as amplified eddy currents, which in turn lead to more severe image distortions and inter‐shot misalignments.

Regarding the acquisition, a pulsed gradient spin echo (PGSE) experiment^20^ combined with EPI readout, typically used in dMRI, requires an additional dead time before the refocusing pulse to meet the spin echo condition. Spiral trajectories^21^ allow for shorter TEs,^22^ resulting in an increase in SNR.^23^ Moreover, spirals use the gradient system more efficiently than EPI and, therefore, for the same effective resolution and undersampling factors, can accomplish the spatial encoding faster than EPI.^24^ However, artifacts arising from field perturbations (e.g., field inhomogeneities,^25^ eddy currents,^26^ anisotropic gradient delays,^27^ and concomitant fields^28^), are more conspicuous in acquisitions performed with a spiral trajectory than compared with EPI. To remedy these limitations independently of the readout trajectory, an expanded encoding model^29^ has been proposed, which includes the static and dynamic field evolution, as well as coil sensitivity maps in the image reconstruction. The dynamic fields therein can be measured accurately using NMR field sensors.^30^, ^31^ Nevertheless, in the context of in vivo dMRI, to date, this framework has been applied primarily to the brain.^22^, ^32^, ^33^

Prostate cancer (PCa) ranks as the most common form of cancer among men worldwide and is one of the leading causes of cancer‐related deaths.^34^, ^35^ dMRI is a key contrast for assessment of PCa as recommended by PI‐RADS,^36^ and recent advances have made significant strides by proposing innovative frameworks to map various microstructure characteristics using (joint relaxation‐)diffusion MRI scans,^37^, ^38^, ^39^, ^40^, ^41^, ^42^, ^43^ improving PCa aggressiveness determination^44^ and likely reducing the need for invasive biopsies.^45^ Therefore, notable efforts are made by the community^46^, ^47^ to address the key issues—low SNR, poor spatial resolution and image distortions—hampering accurate assessment and quantification in prostate dMRI.

In this work, we combine advances in hardware, software and sequence design to address mentioned issues in prostate dMRI.^48^ In our previous work, we demonstrated that utilizing powerful gradients in “imaging below the neck” is safe^49^ and that strong gradients offer a higher contrast‐to‐noise ratio in cancerous lesions compared to clinical gradients.^18^ Here, we combine ultra‐strong gradients, single‐shot spiral readouts and an expanded encoding model reconstruction and demonstrate high *b*‐value prostate dMRI at ultra‐short TEs with high‐depiction accuracy and substantial gain in SNR. Finally, additional scans with a modified spiral readout were acquired to explore submillimeter in‐plane resolution for prostate dMRI.

## 2 METHODS

### 2.1 Data acquisition

#### 2.1.1 Phantom and study participants

An isotropic diffusion phantom developed at the National Institute of Standards and Technology, (NIST)^50^ was used for sequence testing. This phantom consists of an array of thirteen 30‐mL cylindrical vials filled with variable amounts of the polymer polyvinylpyrrolidone (PVP) with mass fractions of 0% (de‐ionized water), 10%, 20%, 30%, 40%, and 50% and resulting diffusivities of 1.127, 0.843, 0.607, 0.403, 0.248, and 0.128 μm^2^/ms at 0°C, respectively.

Ethical approvals for the human imaging part of our work were obtained from the School of Psychology Research Ethics Committee (REC) of Cardiff University and from the REC of the National Health Service (NHS), Wales. One healthy control (51 years, weight: 75 kg, height: 1.68 m) and one patient (53 years, weight: 70 kg, height: 1.61 m) with a prostate tumor with Gleason score, GS,^51^, ^52^, ^53^ 3 + 3 PCa (at the time of diagnosis, 2019, PI‐RADS^36^ score: 3) were scanned after providing written consent. The participants were advised to follow a low residue diet for 24 hours prior to the scanning.

#### 2.1.2 Equipment and protocols

Images were acquired on a 3 T Connectom research‐only scanner (Siemens Healthcare, Erlangen, Germany) with a 56‐cm inner diameter gradient using two surface coils (18‐channel body coil, Body 18, and 32‐channel spine coil, Spine 32, from the same vendor).

Multi‐echo gradient echo (GRE) images were acquired for the estimation of the static $\Delta B_{0}$ map and receiver coil sensitivities. The imaging parameters were: TE = 2.32/4.64/6.96/9.28 ms (for the even echoes), TR = 547 ms, in‐plane resolution = 3.07 × 3.07 mm^2^, slice thickness = 5.0 mm, number of slices = 18, no slice gap, in‐plane FOV = 440 × 260 mm^2^.

**FIGURE 1:**
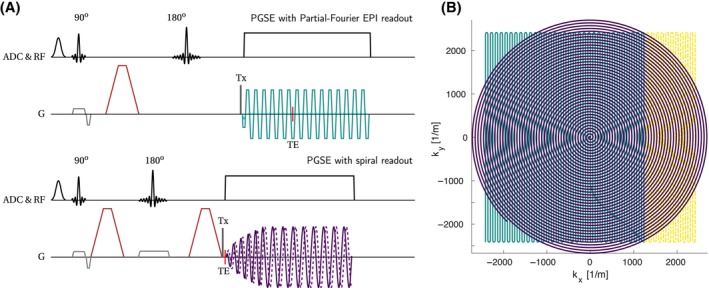
(A) Sketch of the PGSE pulse‐sequences: Single‐shot EPI readout (*top*) and single‐shot spiral readout (*bottom*). The EPI sequence implemented by the vendor has additional navigators for phase correction^57^ played out between the excitation and the first diffusion gradient (*dark gray dashed lines*). ADC (here analog to digital converter) and RF: Spectral fat (Gaussian) saturation, excitation and refocusing pulses and ADC. G: Slice selective and refocusing gradients with crushers/spoilers (*in light gray*), diffusion gradients (*in red*), readout (EPI or spiral *in teal and violet colors*, respectively, matching visualization of trajectories in parametric view, B), and trigger for dynamic field camera (Tx, *in dark gray*). (B) Parametric view of readout trains with matched k‐space area (teal + yellow areas = purple area): Partial Fourier (PF) EPI (*teal line*, *yellow dashed line* indicates PF‐reconstructed points) and spiral (*violet line*).

A PGSE sequence with the flexibility to use arbitrary readout trajectories (Figure 1A, “prototype sequence”) was used to acquire dMRI images along 15 non‐collinear directions distributed on a sphere^54^ at diffusion weightings of b = [0, 0.05, 0.5, 1.5, 2, 3] ms/μm^2^ (maximum gradient amplitude, G_max_ = 247 mT/m, maximum slew rate, SR_max_ = 83.3 T/m/s, diffusion gradient duration, δ = 5.7 ms, diffusion time, Δ = 23.3 ms) and TE of 53 and 35 ms for EPI and spiral, respectively, TR = 3 s, in‐plane resolution = 1.15 × 1.15 mm^2^, slice thickness = 5 mm, and 18 slices, no gap. The timing of the diffusion block was optimized for EPI. EPI and spiral readouts were designed in MATLAB using prototype code^55^ and the Time‐Optimal Gradient Design toolbox,^56^ respectively, and were matched in spatial resolution (i.e., covered k‐space area, including the PF‐reconstructed points for EPI, Figure 1B.). For all dMRI scans, we report resolution as 1/k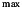, where k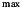 is the radius of the outer circle in the k‐space encompassed by the spiral, that is, 1.15 mm corresponds to 1.30 mm in Cartesian resolution definition. For both readout trajectories: G_max_ = 39 mT/m, SR_max_ = 186 T/m/s, and FOV = 220 × 220 mm^2^. EPI: undersampling factor *R* = 2, partial Fourier (PF) factor = 6/8, phase encoding (PE) = anterior–posterior; Spiral: *R* = 2.24 (*R* of the spiral was adjusted to match the readout length with the EPI: ≈ 44 ms).

Furthermore, dMRI with the vendor's PGSE EPI sequence (“Reference EPI,” in short “Ref. EPI”) was acquired, with all sequence modules matching the ones in the prototype sequence as closely as possible, but additionally including navigators for phase correction.^57^ All data were acquired with the minimum feasible dwell time. Due to limits in the number of samples per segment (where a segment is one k‐space line in the Ref. EPI but the entire readout for the prototype sequence, including the prephaser in case of the EPI), this lead to slight dwell time deviations: Spiral—2.70 μs, EPI—2.80 μs, Ref. EPI—1.6 μs. Each of the dMRI protocols took 4 min, 45 s.

Structural MRI scans were acquired using a 2D T_2_‐weighted turbo spin echo sequence with TE = 97 ms, TR = 4 s, in‐plane resolution = 0.63 × 0.63 mm^2^, slice thickness = 3 mm, number of slices = 23, no slice gap, in‐plane FOV = 200 × 200 mm^2^, PE = right–left. The total acquisition time of the structural scan was 3.5 min.

For the diffusion scans, field dynamics during the readout were monitored in a separate experiment, using a dynamic field‐camera (Skope Magnetic Resonance Technologies AG).^30^, ^31^

#### 2.1.3 Spiral design with varying constraints

The image resolution attainable with single‐shot spiral readout trains is limited by two factors:

1. With increasing readout length and continued slewing of the magnetic field gradients to increasing amplitudes, the risk of peripheral nerve stimulation (PNS) increases.^58^
2. The traversal of a broader frequency spectrum increases the likelihood of hitting one of the hardware resonance bands, which can lead to increased mechanical wear and helium boil‐off.^59^

**FIGURE 2:**
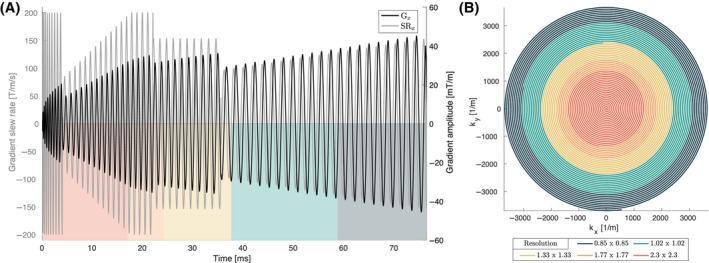
Overview of the parameterization of a spiral readout with varying constraints. (A) The readout gradient and slew rate time courses for a single axis (X‐axis) are depicted. The shaded color‐coded areas match those used to represent different reconstructed resolutions of the k‐space parametric view (B) of the readout. (B) k‐space parametric view showing the k‐space coverage of the readout for a set of reconstructed resolutions, where for the finest resolution, k‐space points depicted by all colors (i.e., full readout trajectory) are included in the reconstruction. Please note that for the readability of the figure, only 5 out of 6 different resolutions that were reconstructed are shown.

For a given coil geometry, the former (1.) can be mitigated by lowering the slew rate throughout the entire readout, however this is at the expense of readout time and it is more economic to lower it only in the affected (later) stages of the readout. The latter (2.) can only be mitigated by avoiding the relevant frequency bands, that is, slowing down the spiral k‐space traversal in the affected k‐space regions.^60^ Therefore, we implemented time‐varying gradient amplitude and slew rate constraints to avoid both hardware resonances on the one hand and exceedance of the PNS limits^61^ on the other. For further details, see Supporting Information S1. In the following, we will refer to this readout as a “spiral with varying constraints.” A dMRI dataset from a healthy participant with 0.85 mm in‐plane resolution using such a spiral was acquired (i.e., full readout trajectory in Figure 2). The maximum PNS tolerance was 96% of the maximum allowed according to the SAFE model,^61^ with 4% buffer, allowing the PNS contributions of sequence modules, other than the readout train, to be tolerated. The length of the spiral readout was 76.43 ms, and it reached G_max_ = 46 mT/m, SR_max_ = 200 T/m/s, with remaining sequence parameters as described in the previous paragraph.

### 2.2 Image reconstruction

The dMRI EPI data acquired with the Ref. EPI sequence were reconstructed using the vendor's GRAPPA‐based reconstruction^62^ with navigator‐based correction^57^ (“reference reconstruction”).

The dMRI data acquired with the prototype sequence were reconstructed using an expanded encoding model^29^, ^63^ (SENSE‐based approach)^64^ including static $B_{0}$‐inhomogeneities, coil sensitivities, and the measured field dynamics (up to 3rd‐order spherical harmonics and 2^nd^‐order concomitant fields^28^, ^65^), using commercially available software (Skope‐i, Skope Magnetic Resonance Technologies). GRE scans were reconstructed using the nominally prescribed trajectories. The static off‐resonance maps and coil sensitivity maps were obtained and processed as described previously.^66^, ^67^ The vendor's correction of $B_{0}$ eddy currents (EC) was reversed prior to feeding the coil data into the reconstruction since the same information is captured (more accurately) in the measured field dynamics.^68^

The spiral with varying constraints was reconstructed for a set of different resolutions, namely 0.85, 1.02, 1.33, 1.77, 2.04, 2.30 mm^2^ by reducing the number of k‐space samples included in the reconstruction accordingly and adapting the reconstruction matrix size. The coarsest resolution recommended in the PI‐RADSv2 guidelines^36^, ^69^ is 2.5 × 2.5 × 4 mm^3^, which corresponds to 25 mm^3^ voxel volume, or 20 mm^3^ with the resolution definition used here. In our study, the biggest voxel volume reconstructed is slightly larger than this, ≈ 27 mm^3^. Figure 2 shows the k‐space parametric view (*left panel*), and time course of the readout gradient amplitude and slew rate (*right panel*) of a single axis (X‐axis). Please refer to the Supporting Information S2 for the analysis of predicted PNS.

### 2.3 Data processing and analysis

dMRI images were corrected for gradient non‐uniformity induced distortions,^70^, ^71^ and spatio‐temporally varying *b*‐matrices^72^ were computed. For qualitative analysis, direction‐averaged signals^73^ were calculated for each shell.

#### 2.3.1 NIST phantom data

The phantom data were used to assess eddy current induced distortions and to evaluate the diffusivity estimation.

Diffusivities were estimated by a mono‐exponential decay for each diffusion direction for all sampled *b*‐values separately. The degree of eddy current‐induced image artifacts was then assessed with a pixel‐wise coefficient of variation (CoV) of diffusivities for a given *b*‐shell.^74^ The CoV was calculated as $\frac{\sigma }{\mu }\cdot 100\%$, where σ is the standard deviation and μ is the mean value of the estimated diffusivity across all directions.

A diffusion tensor representation (DTI) with a non‐linear weighted least squares fit^75^ accounting for the spatio‐temporally‐varying *b*‐matrices, was estimated from the dMRI data (using *b*‐value ≤ 1.5 ms/μm^2^) to evaluate the accuracy and precision of the mean diffusivity (MD) estimation. The voxel‐wise estimates of MD were extracted from within the vials from a single slice and grouped based on the concentration of the PVP. The extraction mask was defined on the *b* = 0 ms/μm^2^ image acquired with either the reference or the prototype sequence using an automatic edge detection algorithm with additional erosion to exclude voxels in the proximity of vial edges. Since for our measurements the phantom was not cooled down to 0°C, we used scaling coefficients as reported in Reference 76 for PVP K30 used in the phantom,^77^, ^78^ to compare the measured MD at 22°C with the reported reference values in the phantom manual.

#### 2.3.2 In vivo data

To evaluate the efficacy of the used $B_{0}$ mapping method for the application to prostate imaging, a gradient map of the raw $B_{0}$ map was calculated and compared with the smoothed $B_{0}$ map and the reconstructed images.

The image misalignment caused by long‐term EC effects was assessed via calculation of edge congruency maps using *b* = 0 ms/μm^2^ images interleaved throughout the diffusion sampling scheme. The edge congruency map was defined as a sum of the binary masks (of each *b* = 0 ms/μm^2^) containing edges of the tissue. The higher the value of the voxel in the congruency map, the more frequently the given voxel was classified as “edge” during automatic edge detection^79^ across repeated *b* = 0 ms/μm^2^ shots.

SNR maps were determined using the “pseudo multiple replica method”^80^ inspired by.^23^ First, the noise covariance matrix was calculated from noise prescans, that is, without gradients and RF transmission,^81^ including in total 1.6 × 105 noise samples. Complex Gaussian noise of the estimated covariance was then added to the raw coil data before image reconstruction. Noise maps were estimated as the pixel‐wise standard deviation of image magnitude over 200 images based on different noise instances. SNR maps were calculated by dividing a magnitude image reconstructed without additional noise by the respective noise maps. The SNR analysis was performed across all directions and all *b*‐values, but only for a single slice to limit computation time. This was done for both the data from the healthy participant and from the PCa patient. Voxel‐wise percent SNR_gain_ of the spiral over EPI was calculated as SNR_gain_ [%] = 100 ⋅ (SNR_spiral_ − SNR_EPI_)/SNR_EPI_, where SNR_spiral_ and SNR_EPI_ are the SNR of the spiral and EPI data, respectively. The median (with interquartile range) SNR and SNR_gain_ was calculated across all voxels within a manually drawn mask on a single slice outlining the prostate gland (“slice‐averaged”). In addition, for the PCa patient, the SNR_gain_ was also assessed in an ROI placed within the cancerous lesion (“lesion‐averaged”).

For quantitative analysis, MD, fractional anisotropy (FA), mean kurtosis (MK), axial kurtosis (AK), and radial kurtosis (RK) were computed,^82^ accounting for the spatio‐temporal b‐matrices in the kurtosis tensor estimation (DKI, using data with *b*‐value ≤ 2 ms/μm^2^).

The datasets at a range of resolutions from the spiral with variable constraints were analyzed quantitatively using DTI as detailed in Section 2.3.1 and using the described SNR analysis.

## 3 RESULTS

### 3.1 Image alignment and MD estimation in a phantom

**FIGURE 3:**
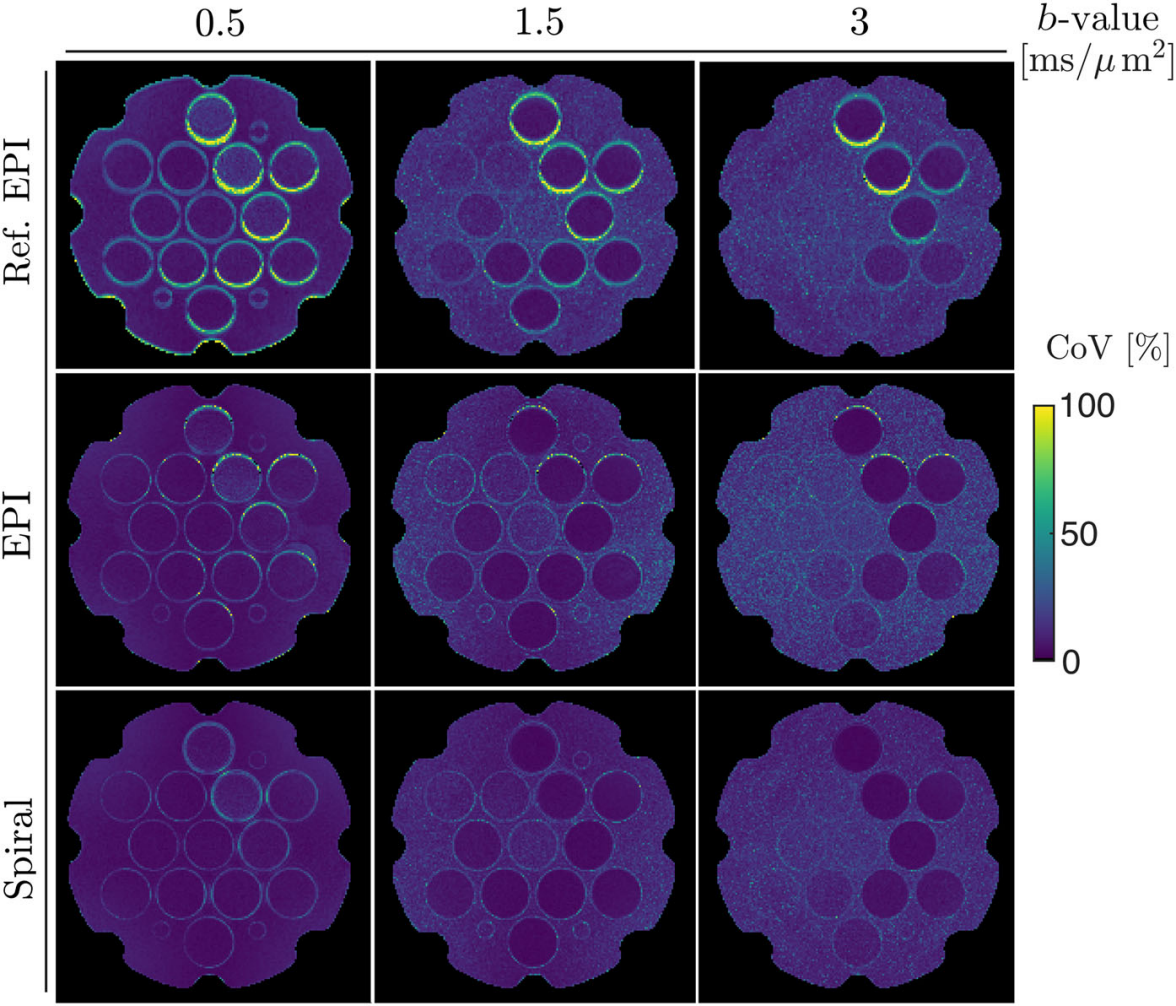
Evaluation of image distortions (e.g., caused by eddy currents) in a diffusion phantom. The CoV was measured across ADC maps estimated per diffusion direction for selected sampled *b*‐values for dMRI data acquired with three PGSE sequences (the vendor's EPI‐sequence—Ref. EPI, as well as the prototype EPI and spiral reconstructed with expanded encoding model).

The CoV maps of diffusivities (Figure 3) for acquisitions with Ref. EPI show much higher values than for acquisitions with the prototype PGSE sequences and image reconstructions based on the expanded signal model. This holds true across all *b*‐values. High CoV values appear in particular on the top and below the vials for Ref. EPI, which is expected since this is the phase‐encoding direction. The observed high CoV values reflect directionally‐dependent geometrical image distortions, which were reduced once the data were reconstructed with the expanded encoding model accounting for dynamic field deviations. For an assessment of the accuracy and bias of MD estimation using DTI applied to the data, please refer to Supporting Information S3.

### 3.2 $B_{0}$ mapping and image congruency of in vivo data

**FIGURE 4:**
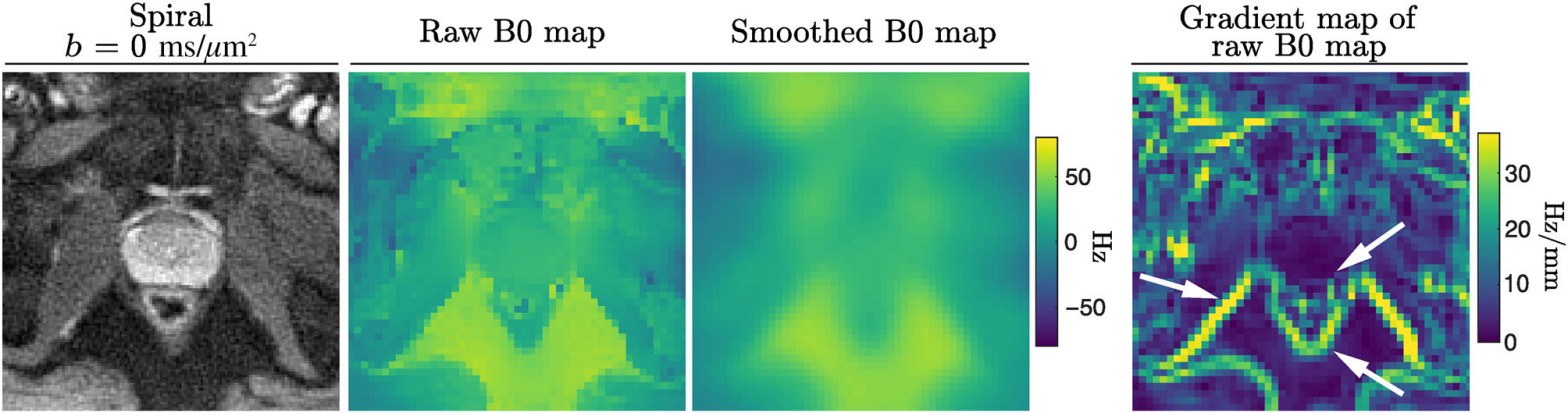
Evaluation of $B_{0}$ mapping method for abdominal imaging in vivo. The *b* = 0 ms/μm^2^ image acquired with spiral readout (*left*), raw and smoothed $B_{0}$ map (*middle*), and magnitude of the gradient map of raw $B_{0}$ map (*right*) are shown. The white arrows point to the areas of abrupt changes in off‐resonance which are not reflected in the smoothed map and therefore cause local blurriness in the respective areas in the reconstructed spiral images.

Figure 4 shows the appearance of the static off‐resonance map for a central slice containing the prostate gland. Whereas the prostate itself exhibits a homogeneous $B_{0}$ distribution, resulting in good visibility of anatomical structures (spiral image in *leftmost panel*), sharp edges occur in some of the surrounding tissues, for example, muscle‐fat interfaces. This is highlighted with white arrows in the gradient map of the raw $B_{0}$ map (*rightmost panel*). Since the smoothed $B_{0}$ map, which enters the image reconstruction does not fully capture these sharp edges, slight blurring can be observed in some of these places. Notably, the interface between prostate and rectum is well recovered.

**FIGURE 5:**
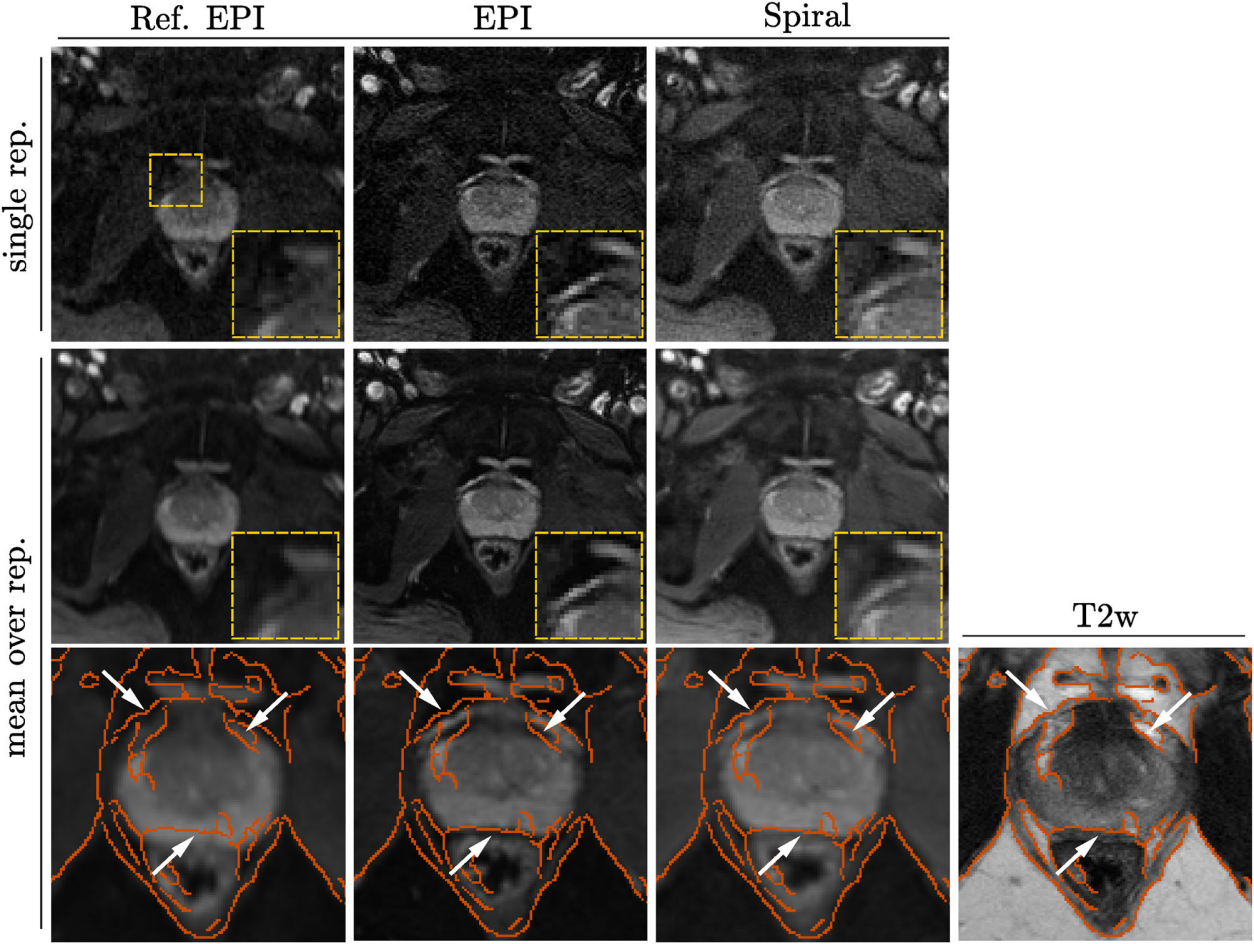
Qualitative evaluation of dMRI images acquired and reconstructed using different methods. A single shot *b* = 0 ms/μm^2^ image (1st in each series of acquisition) from a patient imaged with three PGSE sequences (*top row*): the vendor's EPI‐sequence, i.e., Ref. EPI (*1^st^ column*) and the prototype sequence with EPI and spiral readout (*2^nd^ and 3^rd^ column*). Insets of magnified area of prostate gland are provided showing decreased blurriness in spiral and, the most, in EPI, reconstructed with field monitoring. Average *b* = 0 ms/μm^2^ for the three protocols (*middle row*), and zoomed‐in images with overlaid edges detected on T_2_‐weighted image (*bottom row*). The geometric consistency of fine anatomical features is highlighted with white arrows for the different modalities.

Figure 5 shows single (*top row*) and average (*middle row*) *b* = 0 ms/μm^2^ images acquired with either EPI or spiral readouts, reconstructed using two different approaches (the vendor's software or the expanded encoding model). Tissue edges and other fine anatomical features are better preserved when accounting for field perturbations using the expanded signal model (2^nd^ vs. 1^st^ column), while clearly blurred in Ref. EPI. The signal intensity in the prostate and surrounding muscles is higher at shorter TE (3^rd^ column). In addition, anatomical structures in dMRI with the expanded encoding model reconstructions align better with the morphology in the T_2_‐weighted image.

**FIGURE 6:**
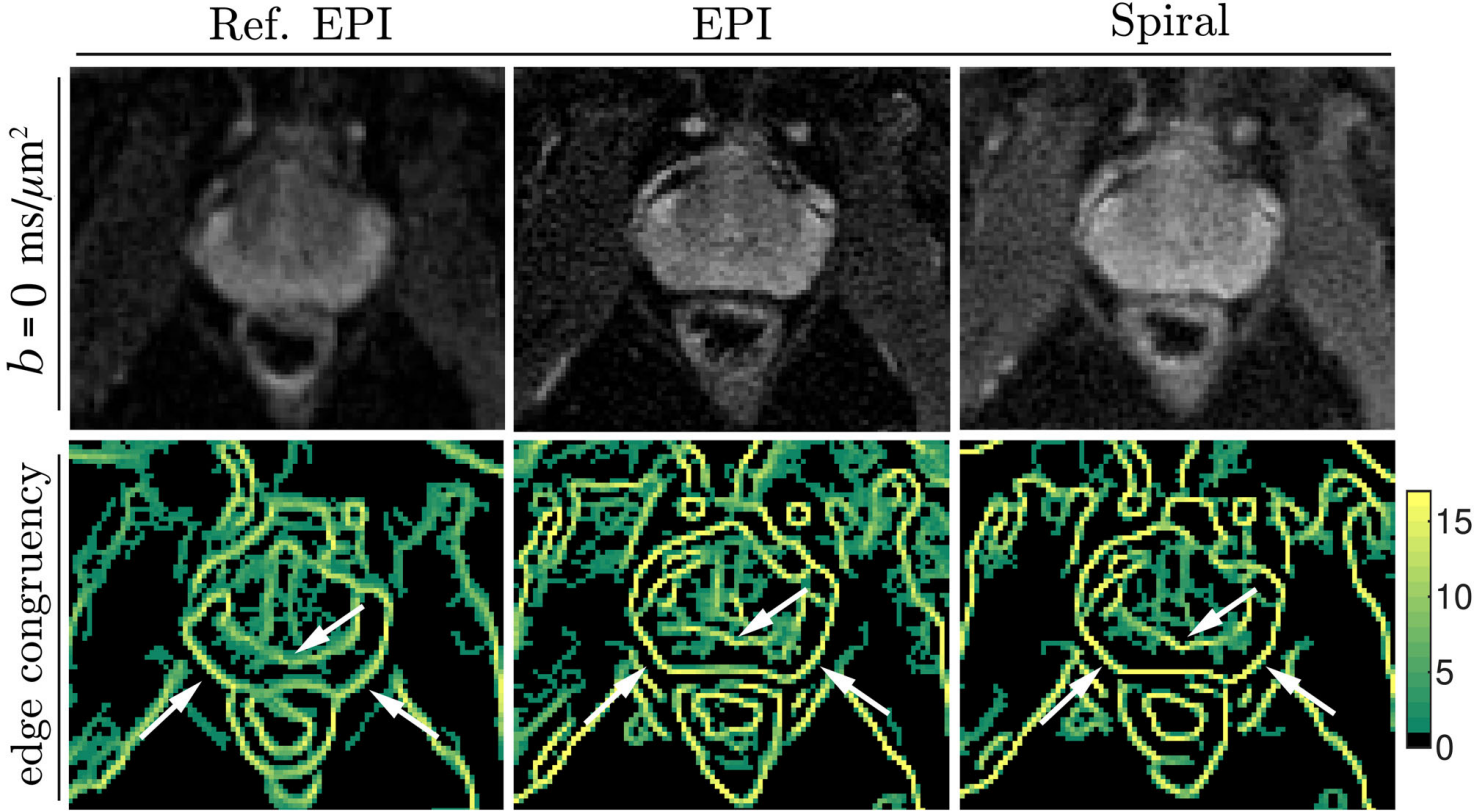
Average *b* = 0 ms/μm^2^ (*top row*) and edge congruency maps (*bottom row*) are shown for three sequences and two different reconstruction algorithms. The scaling of the map was defined by the total number of images at *b* = 0 ms/μm^2^ that were acquired, that is, yellow color with the maximum value of 17 means that the given voxel was detected as being an “edge” voxel across all 17 *b* = 0 ms/μm^2^ volumes. Examples of areas of (in‐)consistent edge detection are highlighted (*white arrows*); for those regions (e.g., the wall between the peripheral zone of the prostate and rectum), the best performance was achieved once the data were reconstructed with the expanded encoding model, regardless of the readout technique employed.

In addition to the geometric consistency of mean *b* = 0 ms/μm^2^ images with the T_2_‐weighted image shown in Figure 5, the edge congruency maps (Figure 6) corroborate a notable improvement in edge consistency across repetitions in data reconstructed with the expanded encoding model. This improvement is particularly evident in distinguishing between the peripheral zone of the prostate and the rectum. For the Ref. EPI acquisition, the edges of the congruency map are more blurred, most likely due to eddy current effects which are incompletely compensated by pre‐emphasis and signal demodulation using the vendor's $B_{0}$ EC model.

### 3.3 SNR analysis

**FIGURE 7:**
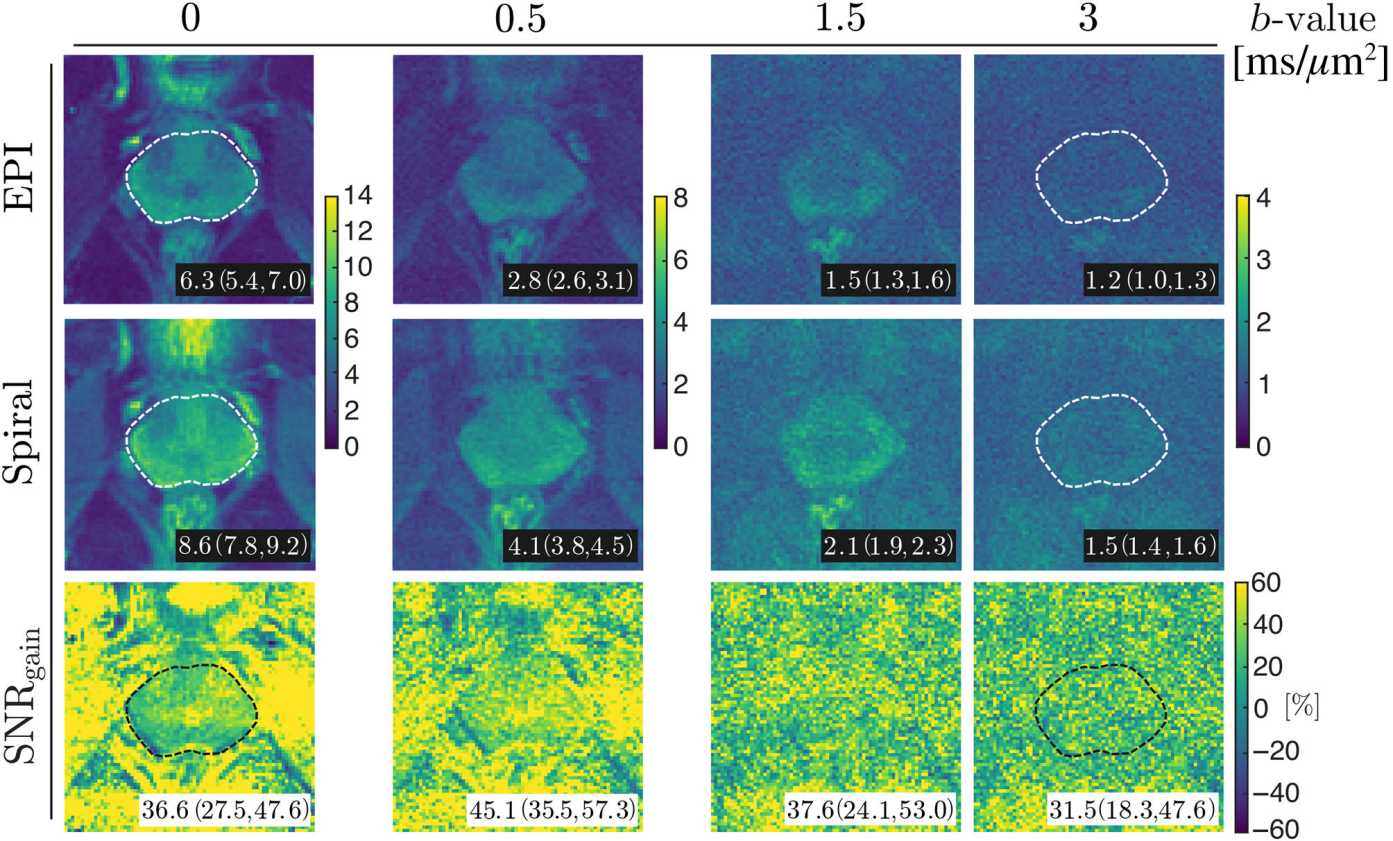
SNR maps (*top row*) of *b* = 0 ms/μm^2^ (*1^st^ column*) and diffusion‐weighted volumes (*2^nd^ to last column*) from a healthy participant dataset obtained with the sequences shown in Figure 1 (PGSE with EPI or spiral readout) and reconstructed using the expanded encoding model. Maps of SNR_gain_ are also presented for each *b*‐value (*bottom row*). Note the different color scales between rows and columns. The values reported in the bottom right corners of the images represent the median with interquartile range of SNR or SNR_gain_ within the prostate mask (*white/black dashed line*). For clarity of the maps, the mask is only displayed in the images corresponding to the lowest and highest *b*‐values.

**FIGURE 8:**
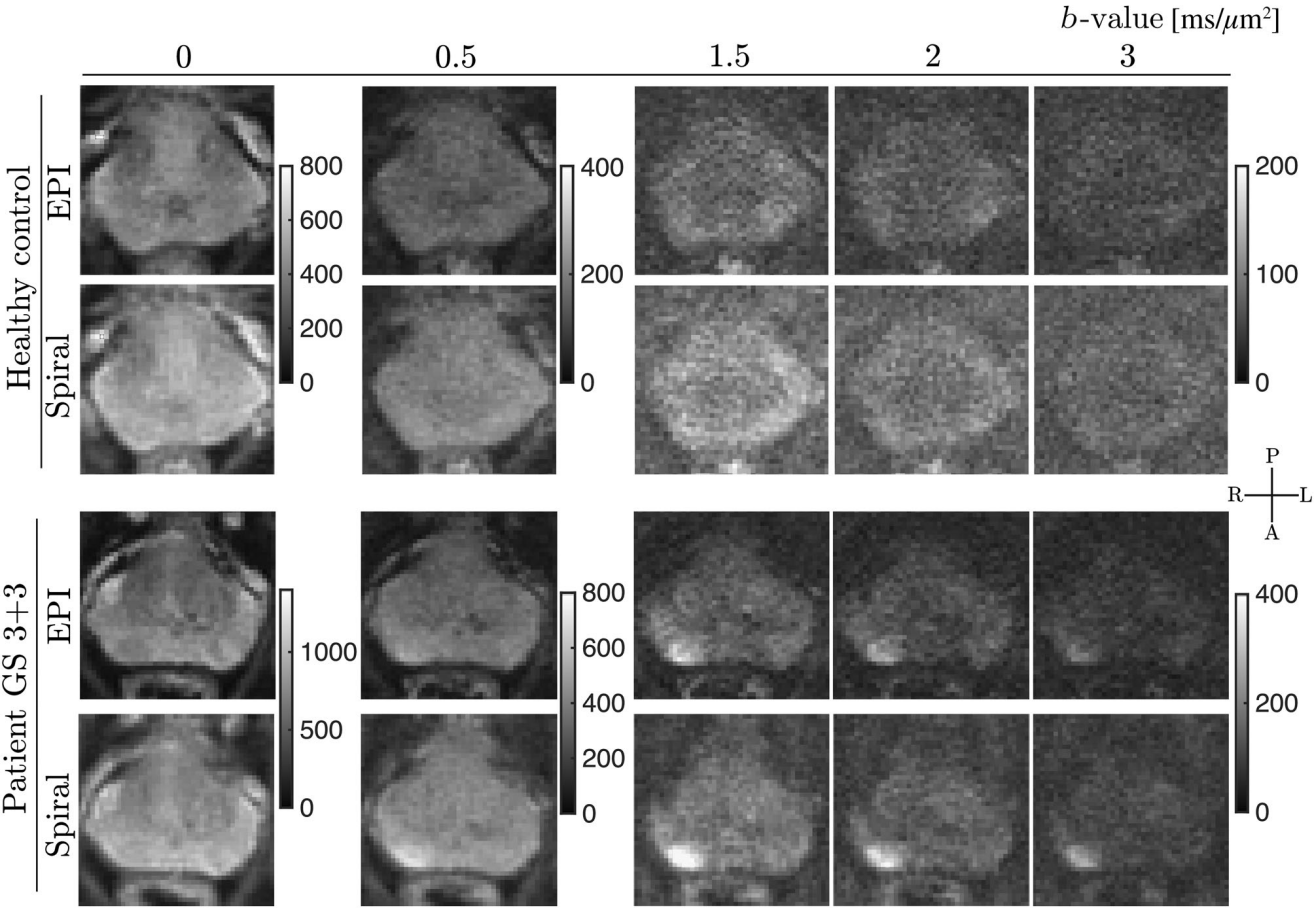
Representative examples of datasets from a healthy control and a prostate cancer patient. Diffusion direction‐averaged signals for selected *b*‐values obtained with the prototype sequence using EPI at TE = 53 ms and spiral at TE = 35 ms are shown (healthy control—*top rows*, prostate cancer patient—*bottom rows*).

The SNR maps (Figure 7) confirm that employing the spiral readout as an alternative to EPI results in higher SNR in dMRI data of the prostate across all *b*‐values. The median SNR_gain_ (with interquartile range given in round brackets) of the spiral readout over EPI ranged between 31% and 45%, depending on the *b*‐value, with the lowest obtained for *b* = 2 ms/μm^2^, and the highest obtained for *b* = 0.5 ms/μm^2^. In the inspected PCa lesion (Figure S3 in Supporting Information S4), the median SNR_gain_ of spiral over EPI ranged between 48% and 62%. Figure 8 shows the results from dMRI experiments from two subjects, namely direction‐averaged signals, in which scans acquired with spiral readout exhibit higher SNR as confirmed by numerical analysis.

### 3.4 Quantitative dMRI of the prostate

**FIGURE 9:**
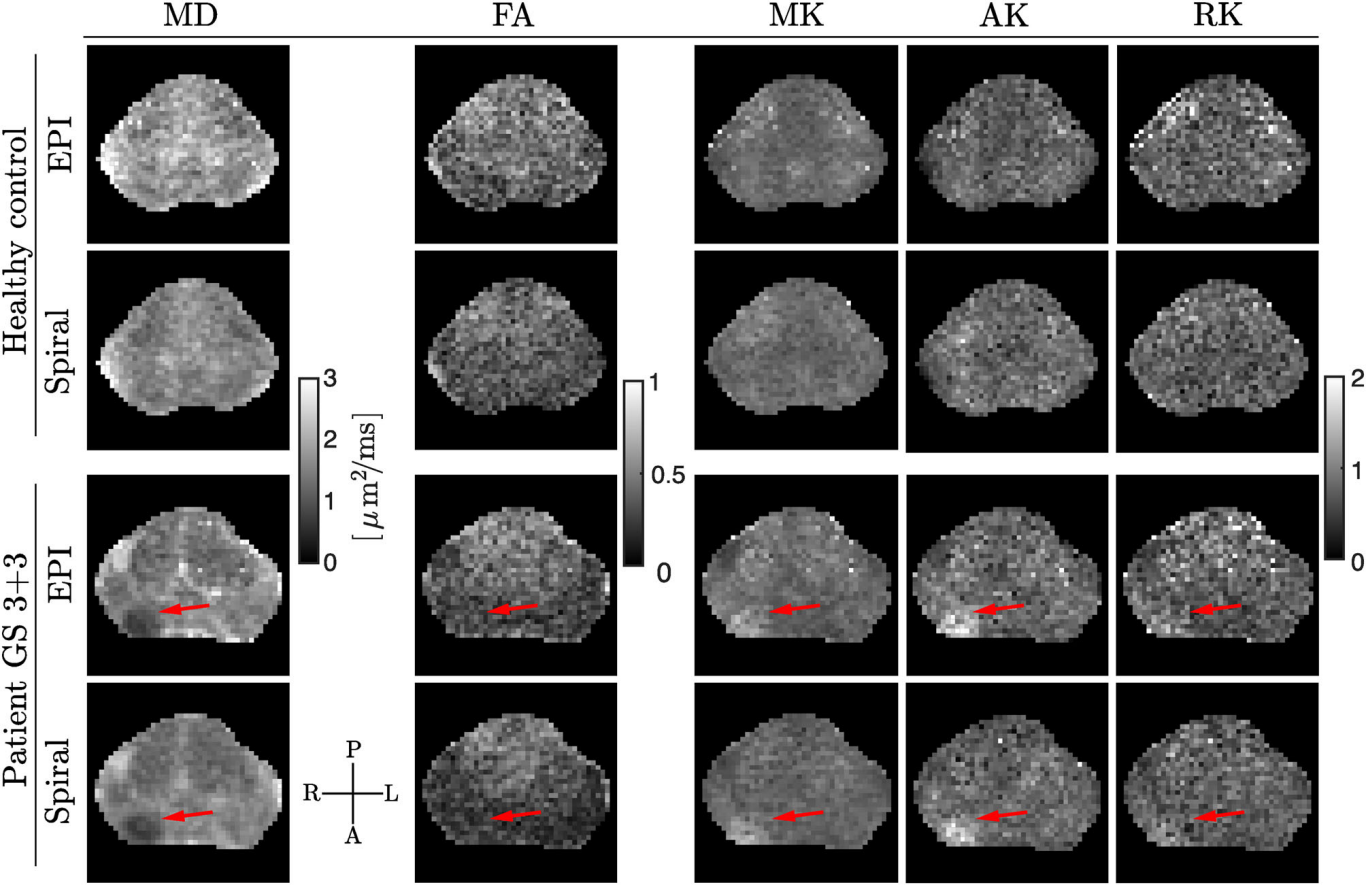
Quantitative maps from DKI were estimated using data from a healthy control (*top*) and a PCa patient (*bottom*), acquired using the prototype PGSE with EPI or spiral readouts. For clarity, the images were masked so as to retain solely the prostate gland. The cancerous lesion (*red arrows*) exhibits lower MD, and higher MK, AK, and (minorly) RK.

Quantitative maps obtained from DKI (Figure 9) show: (i) fine anatomical details consistent with those observed in the T_2_‐weighted image, (ii) more noise‐biased maps obtained from dMRI with EPI than with spirals (e.g., elevated MD, higher FA in the transitional zone), however still excellent conspicuity of the cancerous lesion (i.e., lower MD, higher MK, AK, and RK) regardless of the readout employed, and (iii) clearly distinguishable prostate zones (peripheral and transitional), for example, on the MD map for the patient case.

### 3.5 Prostate dMRI at high in‐plane resolution

**FIGURE 10:**
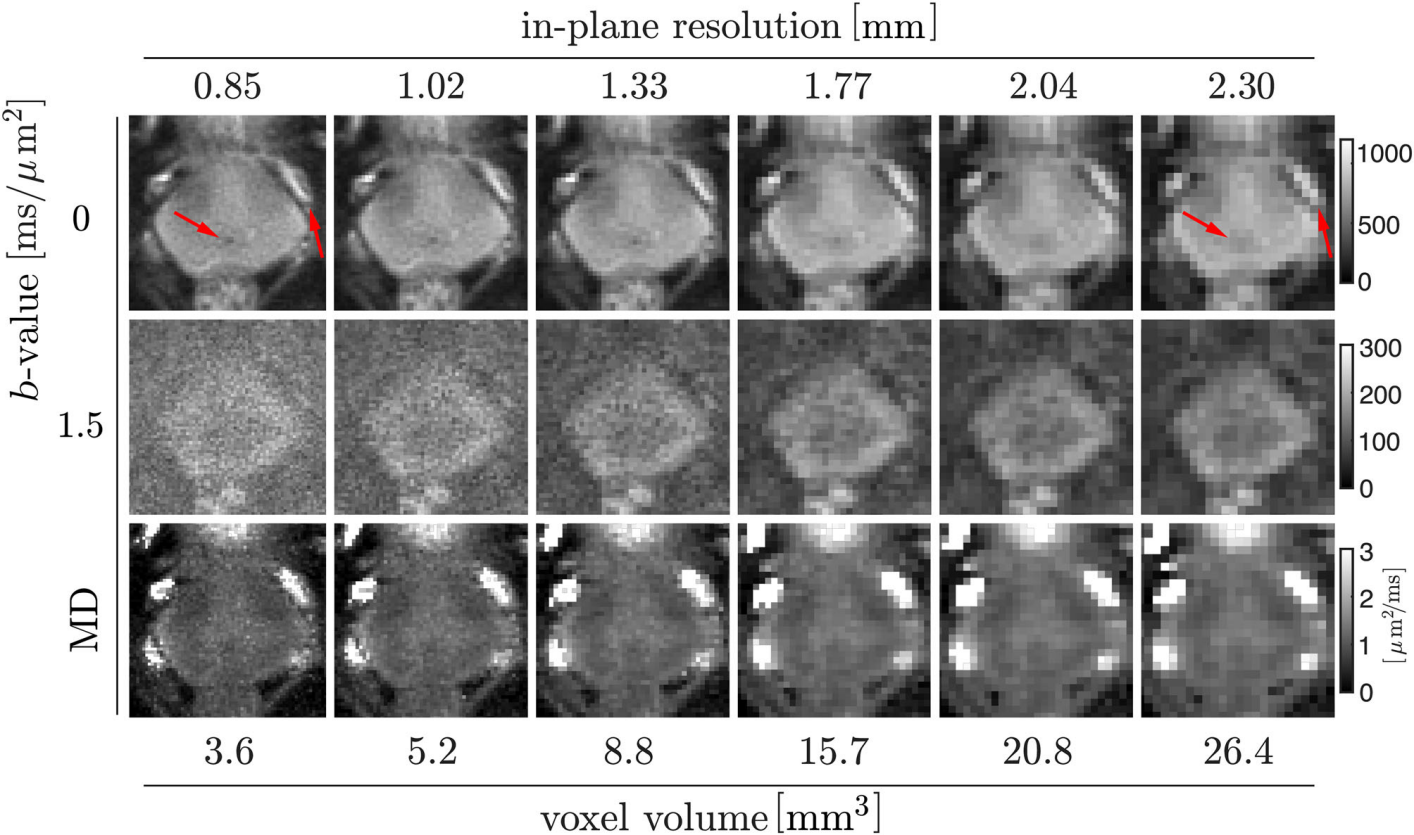
Overview of the diffusion direction‐averaged signals of selected *b*‐values (*1^st^ to 2^nd^ row*) and the estimated MD (*3^rd^ row*) obtained from a healthy control using the prototype sequence with a spiral readout with varying constraints reconstructed at different in‐plane resolutions (*different columns*).

An overview of dMRI data acquired with a spiral with varying constraints trimmed in k‐space to obtain images at a range of different resolutions is shown in Figure 10 (1^st^ to 2^nd^ row). As expected, in the *b* = 0 ms/μm^2^ images with higher resolution, that is, smaller than 1.33 mm in‐plane, fine anatomical features such as periurethral tissue or seminal vesicles remain sharp. The decreased SNR at higher *b*‐value for resolutions smaller than 1.02 mm is a result of ongoing T_2_^*^decay (Figure 10, 2^nd^ row) over the increasing readout length. MD maps derived from the data at lower resolutions are more blurred, making it challenging to outline the zonal anatomy accurately. In contrast, maps at higher resolution exhibit well‐preserved zonal anatomy, despite being visibly noisier. In terms of the estimates, MD maps have similar spatial contrast across different resolutions and are consistent with the values reported for the healthy prostate tissue in group studies.^83^ The SNR analysis using *b* = 0 ms/μm^2^ (reported in Supporting Information S5) confirms the expected behavior of SNR considering its dependence on voxel volume and acquisition time.

## 4 DISCUSSION

We successfully devised advanced field sensing and image reconstruction techniques on a high performance gradient MRI system to image the prostate, resulting in high‐quality dMRI data. By utilizing spirals for spatial encoding, we achieved shorter TEs, leading to an improvement in SNR.^23^

### 4.1 Image reconstruction

The phantom (Figure 3) and in vivo images (Figures 5 and 6) show distortions in the reference reconstructions, which were mitigated in reconstructions using the expanded encoding model.

The CoV evaluation on phantom data allowed for the isolation of hardware‐related effects from anatomical effects (e.g., surrounding tissue of varying T_2_ and diversified intrinsic diffusivities or degrees of anisotropy, contributing to varying degree a direction‐dependent signal in the voxels of interest) and confounding motion effects, which complicate the quantitative evaluation of EC‐induced image distortions in vivo. Instead, the observed high values in CoV maps for Ref. EPI can be mostly attributed to incompletely accounted field perturbations and illustrate the minimum level of image distortions anticipated for in vivo acquisitions employing the same diffusion sampling schemes. The discrepancies of the vial boundaries were minimized with the expanded encoding model reconstruction, leading to very low CoV at the edges of the vials – upon visual inspection even lower than obtained with bipolar or eddy current nulled (ENCODE) gradients (Figure 2 in Zhang et al.^84^).

The analyses presented in Figures 5 and 6, both confirm the observations from the phantom tests. Any residual misalignment in EPI and spiral scans was most likely attributable to motion between repetitions, which was, however, hardly visible during visual inspection of the images. Please note that these analyses—unlike the analysis presented in Figure 3—focus on *b* = 0 ms/μm^2^ images, to facilitate the interpretation by keeping the contrast as close as possible to the reference T_2_‐weighted scan. In in vivo measurements, any diffusion weighting would alter the contrast and confound anatomical comparisons. However, the effects of short‐term eddy currents in strongly diffusion‐weighted volumes and their mitigation are expected to resemble the observations in the phantom measurements.

Notably, these improvements were achieved without increasing the complexity of the waveform, elongating the TE, or incurring penalties in the *b*‐value. However, this comes at the cost of increased complexity of the acquisition setup. In this work, we relied on measurements of field dynamics with a dedicated NMR camera. Alternatively, if such hardware is not available, characterization of the described effects could be partially replaced using other types of trajectory measurements^85^ or gradient response characterization,^86^, ^87^ with the disadvantage of prolonged measurements and limited frequency resolution,^88^ respectively, and thus not providing complete information on occurring field perturbations. Additionally, alternative methods can be employed to address adverse effects from concomitant fields.^89^, ^90^

The static off‐resonance mapping employed in this work,^66^, ^67^ generally yielded good quality $B_{0}$ maps, that is, low level of artefactual distortions or blurring in the images. The minor blurring that is observed for acquisitions using spirals is due to incompletely corrected off‐resonance (Figure 4), as the current smoothing algorithm may locally oversmooth the fine features of the raw $B_{0}$ map with the aim of removing the noise in other areas. So far, the $B_{0}$ map processing has been optimized primarily for brain imaging, for which field mapping $B_{0}$ is relatively simpler: In abdominal imaging, there is a wider diversity of tissue types, that is, fat, muscles, bones, and voids, that are interwoven over the entire imaging FOV. Consequently, susceptibility can vary greatly over short distances. Future work should investigate more involved mapping algorithms, for example, including spatially varying smoothness penalties. Moreover, the subject's movement and peristalsis in the bowel will alter the field with respect to the initially acquired map and render this map less useful for imaging volumes acquired after motion. This could be mitigated by jointly estimating the image and $B_{0}$ map, that is, updating the $B_{0}$ map for each shot.^91^, ^92^

### 4.2 SNR enhancement

The expected SNR_gain_ by shortening TE from 53 to 35 ms (used for EPI and spiral readouts, respectively, in this work) would be 30% assuming monoexponential signal decay with T_2_ = 70 ms as observed in prostate tissue at 3 T.^93^ The observed SNR_gain_ in *b* = 0 ms/μm^2^ images derived in the numerical experiment is slightly higher (37%) and could, apart from a small share that is explained by the slight dwell time discrepancy, be attributed to the more uniform readout time allocation and spatially uniform g‐factor maps for spirals.^23^, ^64^, ^94^

The SNR in dMRI of multi‐compartmental tissues such as the prostate is influenced by the diverse relaxation and diffusion properties of the tissue. To address this, we conducted an SNR experiment using both the healthy control and patient datasets, with aim to evaluate the changes of local SNR, that is, in healthy and cancerous tissues. The slice‐averaged SNR_gain_ values for both participants increase up to *b* = 0.5 ms/μm^2^ and decrease for the highest *b*‐values. This trend could be attributed to short T_2_ compartments of low to intermediate diffusivity contributing more signal at shorter TE. The lesion‐based SNR evaluation in the PCa patient proves the SNR_gain_ to be higher and fairly constant across *b*‐values. The observed differences in the slice‐ versus lesion‐averaged SNR/SNR_gain_ values in data from the PCa patient are the result of ongoing microstructural changes in the diseased tissue, namely increasing contributions of the epithelium, the compartment with the slowest diffusion, to the signal. Please view Supporting Information S4.

To directly compare the EPI and spiral, we kept the timings of diffusion gradients between protocols constant. However, for a PGSE acquisition with a spiral readout, higher SNR_gain_ could be obtained. Specifically, for optimized timings, that is, δ = 7.5 ms and Δ = 15 ms, with remaining gradient parameters—amplitude and slew rate—unchanged, the minimum achievable TE for *b* = 3 ms/μm^2^ is 30 ms, leading to an expected additional SNR increase of ∼10%. However, we aimed to avoid introducing diffusion time dependence in the data by using fixed timings, which were optimized for minimum TE at *b* = 3 ms/μm^2^ with EPI. Please view Supporting Information S6 for SNR and SNR_gain_ evaluation in acquisitions with EPI and spiral readouts at minimized diffusion times, however, at emulated maximum gradient amplitude to 80 mT/m for diffusion encoding performed to evaluate the performance of methods at more common gradient amplitudes.

### 4.3 DKI fit at shorter TEs

In this work, DKI parameter maps from acquisitions with significantly shorter TEs than so far reported in the literature on prostate dMRI, were obtained. A visual improvement of the DKI maps is apparent in the spiral acquisition as a consequence of higher SNR than in EPI. Moreover, the DKI maps estimated from the EPI acquisition may be more biased due to the low SNR (<2)^95^ at the high *b*‐values of 1.5 and 2 ms/μm^2^ (Figure 7).^96^, ^97^ The unaltered value of FA in the PCa lesion could be a result of the averaging effects of microscopic anisotropy at macroscopic scale.^43^

### 4.4 Prostate dMRI at sub‐millimeter in‐plane resolution

High‐resolution dMRI with single‐shot EPI readouts results in long TE and thus inherently low SNR, which is further exacerbated when high *b*‐values, for example, above 1 ms/μm^2^, are necessary. Our results demonstrate that obtaining sub‐millimeter in‐plane resolution with high *b*‐values in a single shot spiral acquisition is feasible, exceeding the protocol specifications for prostate MRI at 3 T in the clinic^69^ and what has been reported in research.^37^

Single‐shot EPI at a matching resolution of 0.85 × 0.85 mm^2^ and with the phase‐encoding steps placed as in the previous comparisons (avoiding the given hardware resonances and complying with the applicable PNS limitations) would require a readout length of 80 ms, that is, 4 ms longer than the spiral with varying constraints shown here. This means that EPI in this scenario loses even more in terms of sampling efficiency compared to the spiral than in the previously investigated cases with conventional spirals, that is, where PNS and hardware resonances could be disregarded. This additional gain is mostly due to the fact that the spiral readout can at least for the sampling of the inner part of k‐space still take advantage of higher frequency bands, whereas the EPI is largely monospectral. The longer acquisition time results in the achievable minimum TE for the given diffusion weighting being 77 ms for PF EPI, that is, 42 ms longer than the respective spiral.

The notable reduction in SNR in the high‐resolution images (Figure 10) can be attributed to increasing noise content of points further out in the k‐space that are being sampled late during the readout, when a significant portion of the signal has decayed (Supporting Information S5). The observed 3.6‐fold decrease of SNR at *b* = 0 ms/μm^2^ images (from the lowest to the highest resolution) aligns with an estimate using voxel volume and sampling time information (≈ 7.3 bigger volume, and ≈ 80% shorter readout length for 2.3 mm in‐plane resolution), which would predict a 3.4‐fold decrease in SNR, but does not take into account ongoing T_2_^*^ decay. The lower SNR at high *b*‐value images could lead to biased estimations and noise enhancement in quantitative parameters in the diffusion analysis. On the one hand, this effect could be mitigated by using multi‐shot variants of EPI and spiral.^98^, ^99^, ^100^, ^101^, ^102^ They can reduce adverse effects from signal dephasing and T_2_^*^ decay,^103^ however, at the cost of acquisition time and image reconstruction complexity to correct for shot‐to‐shot phase fluctuations.^104^, ^105^, ^106^ On the other hand, debiasing and denoising could help to account for inaccuracies in the diffusion parameter estimates, as was shown previously.^107^, ^108^, ^109^

### 4.5 Future work and potential new applications

Our investigation was confined to the exploration of monopolar trapezoidal diffusion gradients. These are commonly utilized in clinical settings. Considering the highly heterogeneous nature of the prostate gland, diffusion encoding schemes beyond these could be used to assess microscopic tissue compartments^42^, ^110^, ^111^ and establish novel biomarkers of clinical value. Furthermore, by utilizing the greater range of TE values that spirals offer for a given *b*‐value, it becomes feasible to assess prostate properties in more extensive diffusion‐relaxation correlation experiments to separate tissue compartmental signals,^38^, ^39^, ^40^, ^112^ which are more strongly correlated with GS and are better predictors of PCa grade than the ADC alone.^113^

This work may also serve as a template for extending these advanced imaging techniques to other body parts, addressing a gap in the current state of diffusion MRI below the neck. Nevertheless, some circumstances affecting imaging experiments are different between different abdominal organs, for example, proximity to cavities (lung or pancreas vs. rectum), movement (lungs, heart), relaxation times of the tissue (heart: T_2_ = 46 ms,^114^ muscles: T_2_ = 30 ms^115^), or magnetophosphenes. All of which will influence whether or how well the technology works (e.g., transverse relaxation times influence attainable resolution in a single‐shot; proximity to cavities influences how faithfully off‐resonances can be measured with a low resolution GRE prescan and benignity of the inverse problem; movement influences both attainable resolution and fidelity of reference maps). Hence, this warrants investigation into translation of these advanced methods to address specific issues and circumstances in dMRI of different organs, for example, heart.^116^

## 5 CONCLUSIONS

Typically, prostate dMRI is performed at moderate *b*‐values, ranging from 0.8 to 1.4 ms/μm^2^ (PI‐RADS 2.1), mainly due to time and hardware constraints of available clinical MR systems. This work is a stepping stone between current clinical and next‐stage high *b*‐value, high SNR, and submillimeter resolution dMRI. Finally, yet importantly, the combination of strong gradients for diffusion encoding with spiral readouts for spatial encoding unlocks sampling at short diffusion times and short TEs and can potentially lead to improved differentiation between cancer, benign changes and healthy tissue. The improved image quality achieved in this work contributes to robustifying MRI as a virtual biopsy, with the potential to enhance patient comfort and alleviate the clinical burden associated with traditional biopsy procedures.

## FUNDING INFORMATION

This work was supported by a Wellcome Trust Investigator Award (096646/Z/11/Z), a Wellcome Trust Strategic Award (104 943/Z/14/Z), an EPSRC equipment grant (EP/M029778/1), and Siemens Healthcare Limited grant to D. K. J. CMWT is supported by a Sir Henry Wellcome Fellowship (215 944/Z/19/Z) and a Veni grant (17331) from the Dutch Research Council (NWO).

## CONFLICT OF INTEREST STATEMENT

F. F. is an employee of Siemens Healthcare GmbH. M. M. studentship was co‐funded by Siemens Healthcare GmbH.

## Supporting information


**Data S1:** Supporting Information.
