# Supplementary material for: Giving the prostate the boost it needs: Spiral diffusion MRI using a high‐performance whole‐body gradient system for high b‐values at short echo times
Source: Magn Reson Med. 2024 Nov 4;93(3):1256–72. doi: 10.1002/mrm.30351 (PMC11680736; doi:10.1002/mrm.30351)
Supplement: Supplementary file 1 — Data S1: Supporting Information. [file MRM-93-1256-s001.pdf]

Supporting Information for manuscript
"Giving the prostate the boost it needs: Spiral diffusion MRI using a high-performance whole-body gradient system for high b-values at short echo times"

Malwina Molendowska[1,2*], PhD, Lars Mueller[3], PhD, Fabrizio Fasano[4,5], Derek K. Jones[1], PhD, Chantal M. W. Tax[1,6**], PhD, Maria Engel[1**], PhD

1. Cardiff University Brain Research Imaging Centre (CUBRIC), Cardiff University, Cardiff, United Kingdom
2. Medical Radiation Physics, Clinical Sciences Lund, Lund University, Lund, Sweden
3. Leeds Institute of Cardiovascular and Metabolic Medicine, University of Leeds, Leeds, United Kingdom
4. Siemens Healthcare Ltd, Camberly, United Kingdom
5. Siemens Healthcare GmbH, Erlangen, Germany
6. Image Sciences Institute, University Medical Center Utrecht, Utrecht, The Netherlands

* Corresponding author:
Name: Malwina Molendowska
Department: School of Psychology
Institute: Cardiff University Brain Research Imaging Centre (CUBRIC), Cardiff University
Address: Maindy Road, CF24 4HQ, United Kingdom
E-mail: molendowskam@cardiff.ac.uk
**These authors contributed equally to this work.

Supporting Information

S1: Spiral with varying constraints - theory

For the "spiral with varying constraints", the algorithm proposed by Lustig et al. (56) is adapted to use time-varying gradient amplitude and gradient slew rate limits ($G_{\mathrm{max}}(t)$ and $SR_{\mathrm{max}}(t)$ respectively, t being the time that elapses during the readout), rather than global limits.

Avoidance of PNS

Initially, the un-modified algorithm by Lustig et al. is run, using the hardware (HW) limits $G_{\mathrm{max}}^{\mathrm{HW}}$ and $S_{\mathrm{max}}^{\mathrm{HW}}$ as global input constraints for gradient amplitude and gradient slew rate. For a spiral-out trajectory, this results in gradient waveforms where the sum of squares of both the amplitude and slew rate are continuously increasing or staying constant, but never decreasing.

From this gradient time course, the PNS level is predicted (61) which is consequently also monotonically increasing (apart from periodic fluctuations mainly due to asymmetries in PNS generation along the different gradient axes; in case of symmetry across axes, those are much smaller). t_0 is identified as the time point from which onwards the PNS level exceeds the targeted/allowed level. Subsequently, a new gradient time course is created where the input $S_{\mathrm{max}}(t)$ is lowered by a manually chosen amount (e.g., 5%) for $t > t_0$. The new gradient waveforms are checked to not exceed the allowed/targeted PNS.

In the following, the two sections ($t < t_0$ and $t > t_0$) are treated separately.

Avoidance of resonance bands

The curvature of the k-space trajectory at a given point along an Archimedean spiral can be approximated with a circle of the same radius R as the point's distance to the origin. For a single k-space axis (e.g., x), the Cartesian coordinate of such a circle (in polar coordinates, (R, ϕ)) is:

$$k_{\mathrm{x}} = R\,\sin(\phi)\,. \tag{1}$$

Travelling with constant speed along the circle renders ϕ time dependent, $\phi = \omega t$, with angular velocity ω and so $k_{\mathrm{x}} = k_{\mathrm{x}}(t)$. Gradient and slew rate time courses follow as time derivatives:

$$G_x(t) = \frac{1}{\gamma}\frac{\mathrm{d}k_x}{\mathrm{d}t} = \frac{\omega R}{\gamma}\cos(\omega t) = G_{\mathrm{amp},x}\cos(\omega t)\,, \tag{2}$$

$$S_x(t) = \frac{\mathrm{d}G_x}{\mathrm{d}t} = -\frac{\omega^2 R}{\gamma}\sin(\omega t) = -S_{\mathrm{amp},x}\sin(\omega t)\,, \tag{3}$$

with the gradient amplitude $G_{\mathrm{amp,x}} = \omega R/\gamma$ and gradient slew rate amplitude $S_{\mathrm{amp,x}} = \omega^2 R/\gamma$.

In the following, we will move from the description of pure circles to spirals. Realisation of a spiral with maximum speed on a given gradient system with maximum available - that is considering hardware and other constraints - gradient amplitude and slew rate being G_{lim} and S_{lim} (note, the subscript lim denotes limited), respectively results in two modes: In the inner part of the k-space (smaller curvature, k-space acceleration in case of spiral-out), the gradients are in gradient slew-rate limit ($S_{\mathrm{amp,x}} = S_{\mathrm{lim}}$). In the outer part (larger curvature, maximum k-space speed), they are in gradient amplitude limit ($G_{\mathrm{amp,x}} = G_{\mathrm{lim}}$). The transition (denoted as subscript *tra*) occurs at:

$$R_{\mathrm{tra}} = \gamma\frac{{G_{\mathrm{lim}}}^2}{S_{\mathrm{lim}}}. \tag{4}$$

For both modes, the k-space radius can be expressed as a function of frequency f $(= \frac{\omega}{2\pi})$:

- Gradient amplitude limit, $R > R_{\mathrm{tra}}$:

$$R(f) = \frac{\gamma G_{\mathrm{lim}}}{2\pi f} \tag{5}$$

- Gradient slew-rate limit, $R < R_{\mathrm{tra}}$:

$$R(f) = \frac{\gamma S_{\mathrm{lim}}}{(2\pi f)^2} \tag{6}$$

To avoid the frequencies between f_1 and f_2 with $f_1 > f_2$, the constraints in the affected k-space section have to be adapted:

- Gradient amplitude limit, $R > R_{\mathrm{tra}}$:

$$G_{\max}(R) = \frac{2\pi f_2 R}{\gamma} \quad \text{for } R(f_2) > R(f) > R(f_1) \tag{7}$$

- Gradient slew-rate limit, $R < R_{\text{tra}}$:

$$S_{\max}(R) = \frac{R}{\gamma}\left(2\pi f_2\right)^2 \quad \text{for } R(f_2) > R(f) > R(f_1) \tag{8}$$

Note that if a slew rate reduction for PNS mitigation as described above was performed initially, the constraint adaptation for resonance avoidance has to be performed separately for the two sections $t < t_0$ and $t > t_0$ where S_{lim} denotes the PNS-adapted slew rate limit, which is in that case smaller than $S_{\max}^{\text{HW}}$. In contrast, it is always $G_{\text{lim}} = G_{\max}^{\text{HW}}$. However, note that in the case presented here, the gradient amplitude limit is never reached.

S2: Predicted PNS level and frequency content of the spiral with varying constraints

For the spiral readout with varying constraints used in this manuscript, we provide the predicted PNS level (Figure S1) over the time course of the readout. The estimation was performed with the use of the publicly available implementation of the SAFE model (`https://github.com/filip-szczepankiewicz/safe_pns_prediction`, SHA-1 hash d28369da).

Analysis of the power spectra of G_{RO} shows a reduced power content in the resonance bands of the hardware. The visualisation of this cannot be provided as any information about resonance bands is proprietary information of the system's producer.

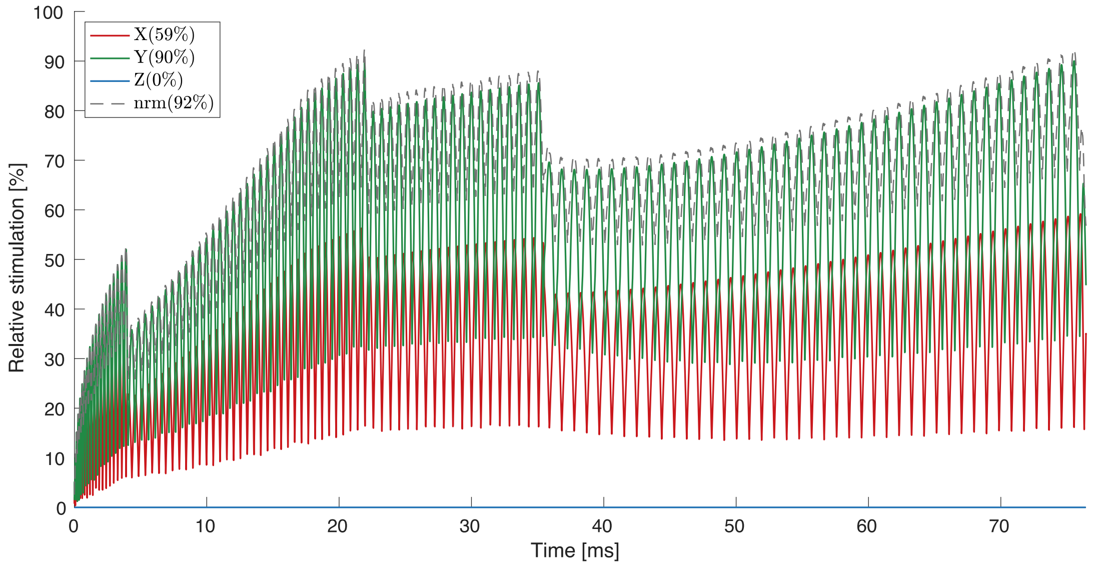

Figure S1: Spiral readout with varying constraints: Predicted PNS as percentage of the maximum allowed level for the trajectory presented in Figure 2 of the main manuscript.

S3: Accuracy and precision of diffusivity estimation in the NIST phantom

Figure S2 shows the results of the MD estimation in PVP solutions for different concentrations. Minor differences in accuracy are observed between EPI and spiral readouts using the prototype sequence. On the contrary, the bias is increased in MD estimates using the data from the acquisition with Ref. EPI. The accuracy of the estimates (narrower distributions of parameter values) is progressively improved with increasing concentration of PVP, with the spiral acquisition performing supremely across all PVP concentrations. Importantly, some of the observed bias could be attributed to the scaling values applied in the analysis.

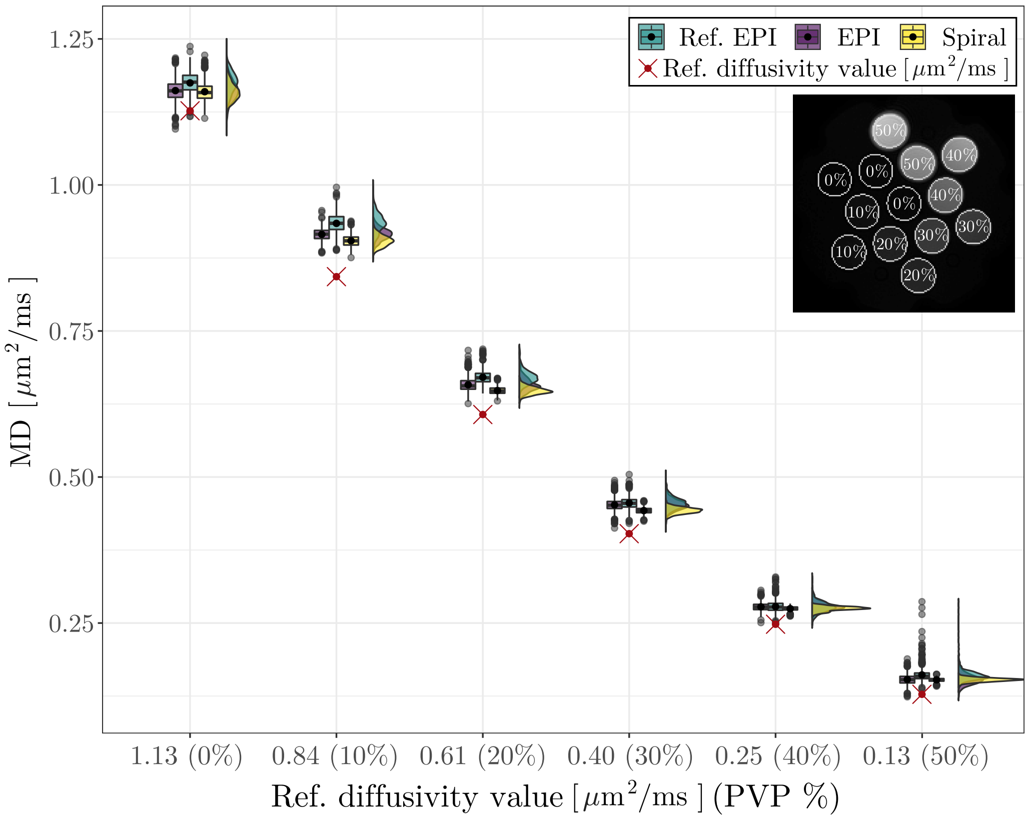

Figure S2: Mean diffusivity (MD) estimates of PVP solutions in the diffusion phantom for Ref. EPI, EPI, and spiral readout. The phantom image (upper right corner) shows the masks (*white circles*) used to extract the MD values for the respective PVP concentration. Box plots (mean, standard deviations, and outliers - *black points*) and density plots of the estimated values are displayed in the main panel, as well as the ground truth values (*red cross*).

S4: SNR analysis of prostate dMRI in PCa patient

Figure S3 shows the SNR and SNR_{gain} maps with the summary statistics (median and IQR) performed using PCa patient data. The following values report the summary statistics of SNR in a ROI defined in the PCa lesion for a vector of b-values = [0, 0.05, 0.5, 1.5, 2, 3] ms/µm^2: [14.9 (14.5, 16.1), 12.6 (12.1, 13.5), 9.0 (8.4, 9.6), 5.0 (4.7, 6.0), 4.4 (3.9, 4.6), 3.1 (2.7, 3.5)] for spiral, and [9.7 (9.2, 9.8), 8.0 (7.6, 8.4), 5.5 (5.3, 6.2), 3.3 (2.9, 3.8), 2.9 (2.6, 3.2), 1.8 (1.8, 2.3)] for EPI, which corresponds to an SNR_{gain} of spiral over EPI that amounts to [60.6 (52.9, 66.7), 59.0 (48.3, 66.0), 61.8 (52.2, 68.3), 60.1 (46.5, 68.1), 48.4 (35.4, 60.4), 62.1 (48.8, 81.2)] %.

The slice-averaged median values of SNR and SNR_{gain} are consistent across both - healthy control (Section "SNR and tissue contrast analysis" of the main manuscript) and patient - datasets. However, for the lesion, we find approximately 2 (1.7-2.2) times higher SNR values across b-values from 0.5-3 ms/µm^2 for spiral acquisitions. For EPI, this increase is slightly

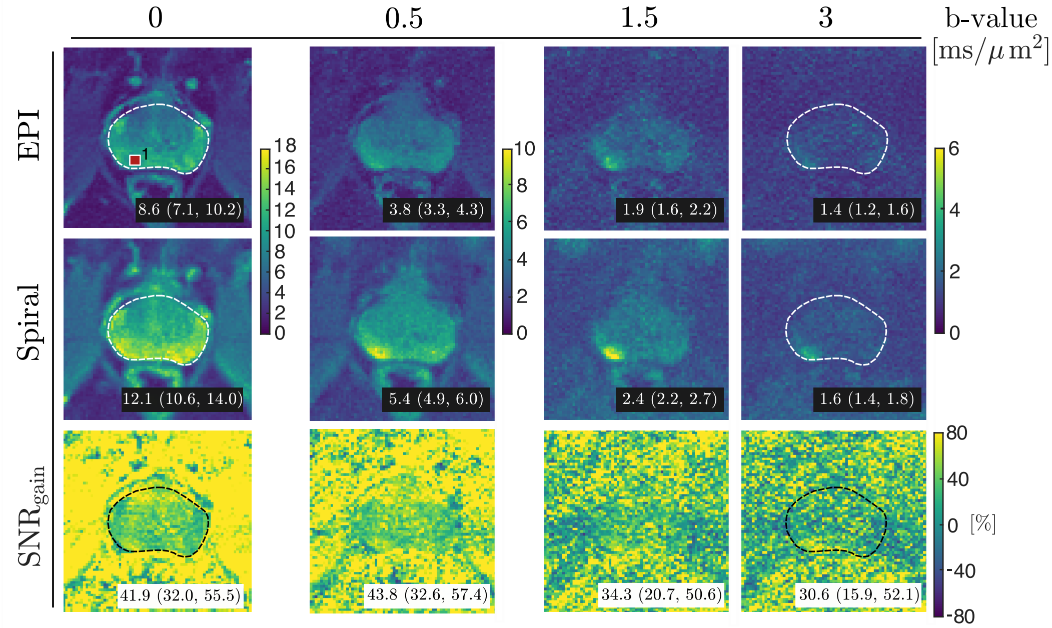

Figure S3: SNR maps (*top row*) of b = 0 ms/µm^2 (*1st column*) and diffusion-weighted volumes (*2nd to last column*) from a patient dataset obtained with the sequences shown in Figure 1 (PGSE with EPI or spiral readout) and reconstructed using the expanded encoding model. SNR_{gain} maps when using spirals instead of EPI are also presented for each b-value (*bottom row*). Note the different colour scales between rows and columns. The values reported in the bottom right corners of the images represent the median with an interquartile range of SNR or SNR_{gain} within the prostate mask (*white/black dashed line*). For clarity of depiction, the mask is only shown in the images corresponding to the lowest and highest b-values.

lower and is within a range of 1.3-1.7. The SNR_{gain} values in the lesion are consistent across b-values.

S5: SNR analysis of spiral acquisitions with varying constraints

Figure S4 shows the SNR maps of the analysis performed using data acquired in a healthy control with a spiral with varying constraints. Slice-averaged SNR values (median and IQR) are shown in Figure S5. For the $b = 0$ s/μm^2, there is approximately a 3.5-fold SNR decrease in the images at the finest resolution, 0.85×0.85 mm^2, with respect to 2.3×2.3 mm^2. The SNR of high b-value dMRI data is close to a low SNR cut-off value of 2 [95]: For $b = 1.5$ s/μm^2, the SNR is higher than 2 for resolutions bigger than 1.33×1.33 mm^2, whereas for $b = 3$ s/μm^2 the SNR values across all resolutions are lower than 2.

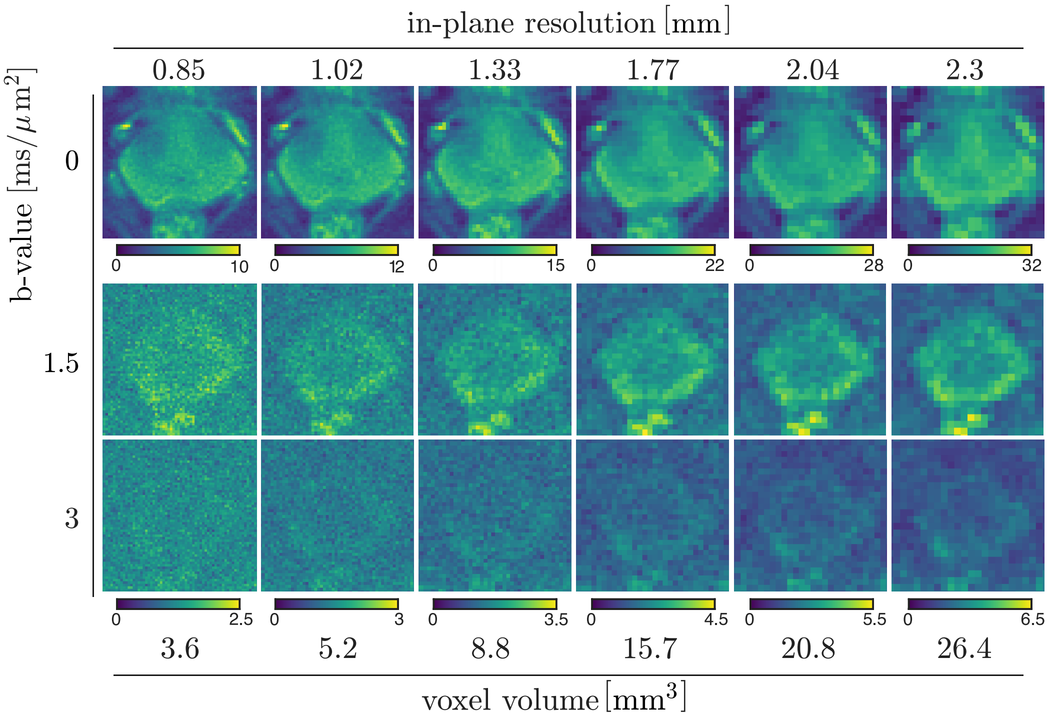

Figure S4: SNR maps of $b = 0$ ms/μm^2 (*top row*) and diffusion-weighted volumes of $b =$ [1.5, 3] ms/μm^2 (*2nd and 3rd row*) from a healthy control dataset obtained with the sequences shown in Figure 1 (PGSE spiral with varying constraints) and reconstructed using the expanded encoding model across different resolutions. Note the different colour scales between rows and columns.

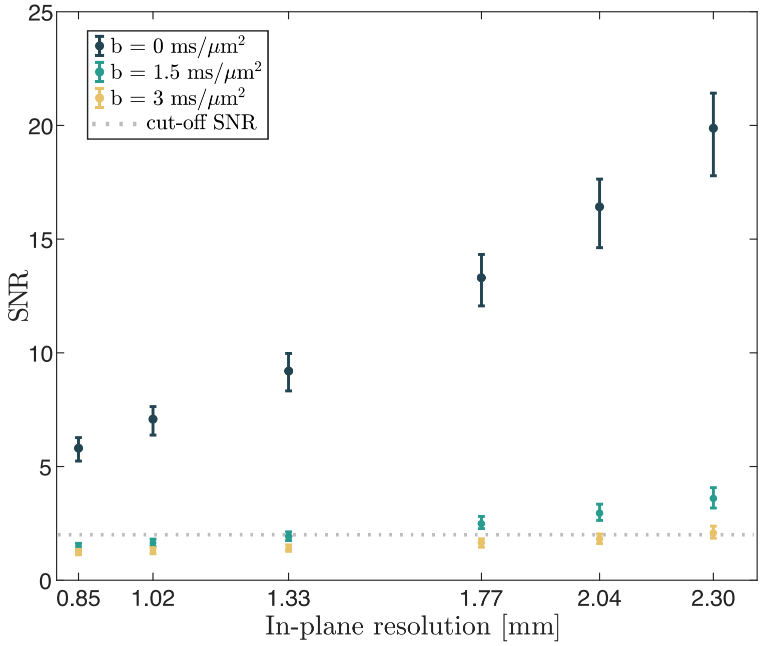

Figure S5: The summary statistics (median with an interquartile range) of SNR within a defined prostate mask for each image resolution. The dashed grey line represents the low SNR cut-off value (116).

S6: SNR and tissue contrast analysis of acquisitions with EPI and spiral readouts at emulated maximum gradient amplitude to 80 mT/m for diffusion encoding

To further highlight the benefits of spirals, we show an analysis of tissue contrast (TC) and SNR_{gain} for a dataset acquired using diffusion protocols (with EPI and spiral readout) based on prototype sequence as compared in the main study but employing G_{max} = 80 mT/m for the diffusion gradients. The parameters of the three new protocols (P) are given below:

- P1. EPI: TE = 68 ms, Δ = 32.8 ms, δ = 15.2 ms. Please note, for this acquisition we removed the time needed for the phase navigators which are present in Ref. EPI protocols; therefore, the achieved TE is shorter by 3 ms in comparison to one achievable with Ref. EPI at 80 mT/m.
- P2. Spiral: TE = 53 ms, Δ = 32.8 ms, δ = 15.2 ms.
- P3. Spiral at minimum TE: TE = 48 ms, Δ = 25.0 ms, δ = 18.6. Please note, for this acquisition we minimised the diffusion encoding time such that maximum b-value reached is 3 ms/μm^2.

Please note that P3 protocol was provided only for the purpose of this supplementary information for the additional insight on effects of minimised TE on TC and SNR_{gain}. In addition, we did not acquire Ref. EPI with diffusion encoding at 80 mT/m as in this experiment we solely want to evaluate SNR and SNR_{gain}, without assessment of geometric fidelity.

One patient (73 years, weight: 89 kg, height: 1.67 m) with a prostate tumour with Gleason score, GS 3+3 PCa (at the time of diagnosis, 2021, PI-RADS score: 5+3) was scanned after providing written consent.

The data analysis was performed as reported in the Methods section of the main manuscript.

The SNR and SNR_{gain} results (Figure S7 and S8) confirm the advantages of spiral readouts over EPI, and indeed, support their utility on MR systems with clinical gradient strengths. For a region of interest defined in midgland of the prostate, the median SNR_{gain} of the spiral readout over EPI ranged between 26% and 43%, depending on the b-value. These values match the results for protocols using 247 mT/m, reported in the main manuscript. However, note that a direct comparison is difficult to draw as the data come from two different patients with underlying tissue properties. If the TE is further minimised in the PGSE with spiral readout (P3), further SNR increases are obtained with preserved gain of TC with respect to EPI (S8).

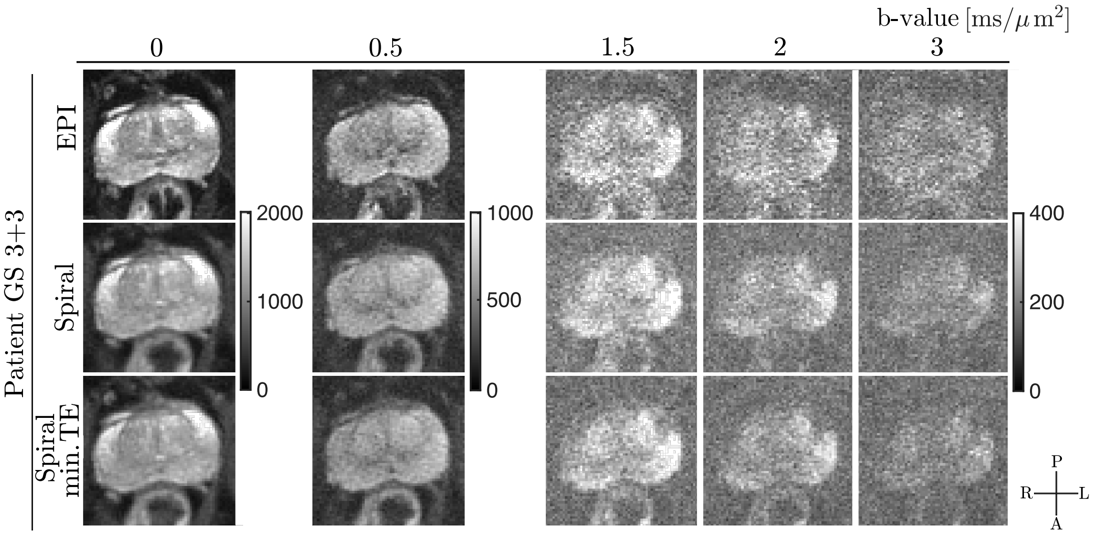

Figure S6: Example of datasets from a prostate cancer patient acquired using adapted protocols from our work, where the diffusion encoding was optimised to keep G_{max} = 80 mT/m, and EPI and spiral readouts; an additional protocol was prepared and acquired, but with spiral at minimum TE. Diffusion direction-averaged signals for selected b-values: EPI at TE = 68 ms, spiral at TE = 53 ms are shown, and spiral at minimum TE = 48 ms.

.

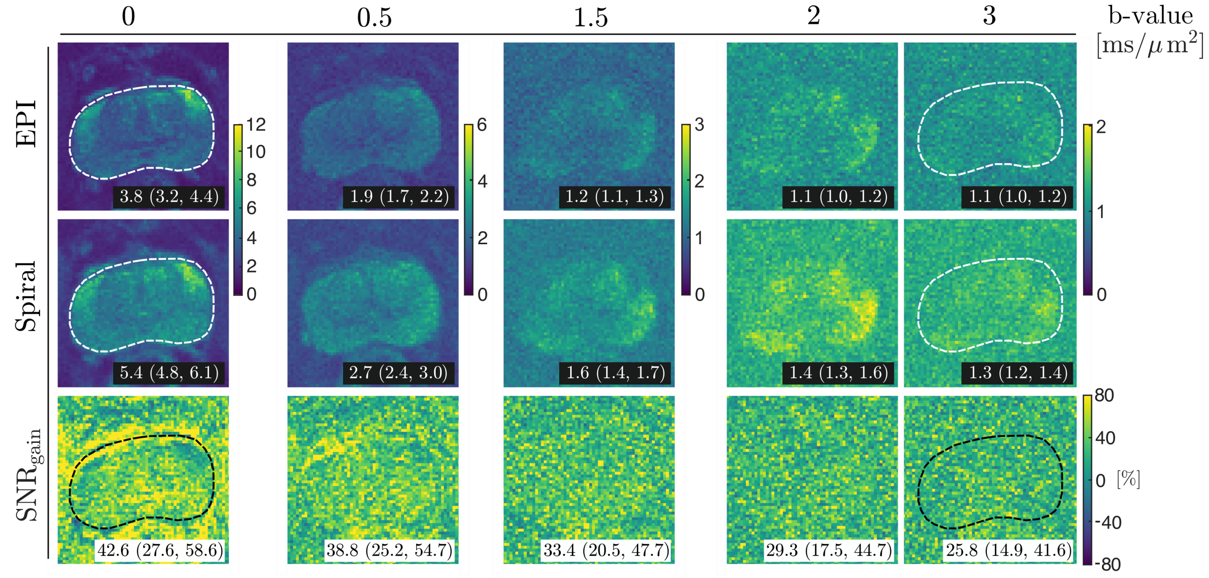

Figure S7: SNR maps (*1st and 2nd row*) of $b = 0$ ms/μm^2 (*1st column*) and diffusion-weighted volumes (*2nd to last column*) from a patient dataset obtained with the adapted protocols using prototype sequence (PGSE with EPI or spiral readout) and reconstructed using the expanded encoding model. The maximum gradient amplitude in the diffusion encoding block was 80 mT/m. SNR gain maps when using spirals instead of EPI are also presented for each b-value (*3rd row*). Note the different colour scales between rows and columns. The values reported in the bottom right corners of the images represent the median with an interquartile range of SNR or SNR_{gain} within the prostate mask (*white/black dashed line*).

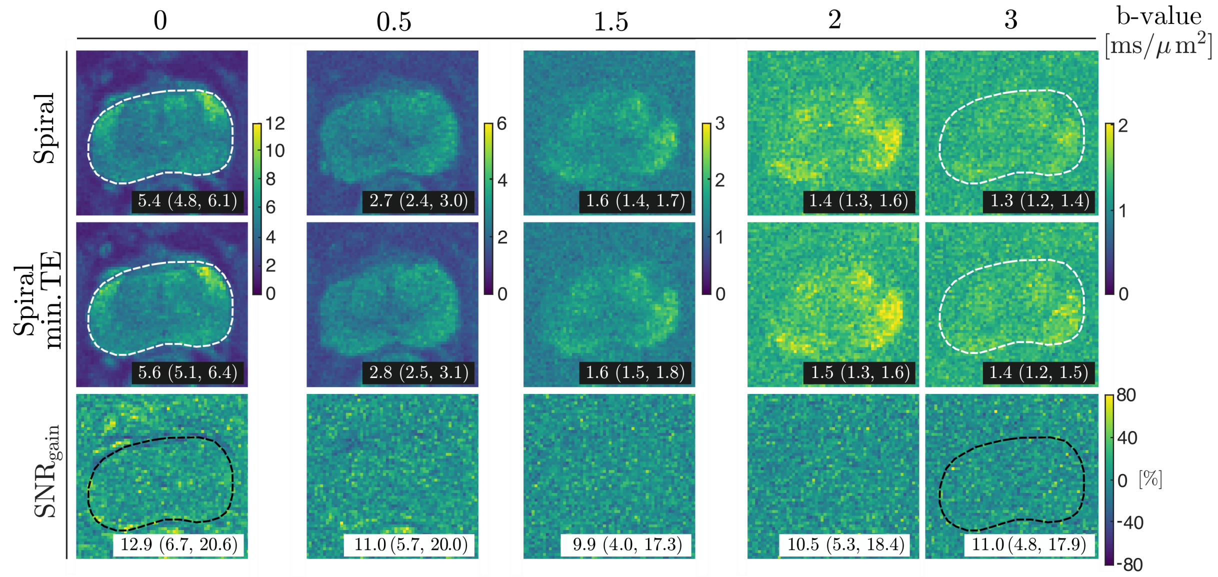

Figure S8: SNR maps (*1st and 2nd row*) of b = 0 ms/μm^2 (*1st column*) and diffusion-weighted volumes (*2nd to last column*) from a patient dataset obtained with the adapted protocols using the prototype sequence (PGSE with spiral readout with different timing of diffusion encoding block and G_{max} = 80 mT/m, please see above) and reconstructed using the expanded encoding model. SNR gain maps when using spirals at minimum TE instead of the one with the diffusion block timings matched to the EPI protocol are also presented for each b-value (*3rd row*). Note the different colour scales between rows and columns. The values reported in the bottom right corners of the images represent the median with an interquartile range of SNR or SNR_{gain} within the prostate mask (*white/black dashed line*).
